# Prebiotic replicase evolution in a surface-bound metabolic system: parasites as a source of adaptive evolution

**DOI:** 10.1186/1471-2148-8-267

**Published:** 2008-09-30

**Authors:** Balázs Könnyű, Tamás Czárán, Eörs Szathmáry

**Affiliations:** 1 Institute of Biology, Eötvös University, Pázmány P. sétány 1/c, H-1117 Budapest, Hungary; 2 Theoretical Biology and Ecology Research Group, Eötvös University, Pázmány P. sétány 1/c, H-1117 Budapest, Hungary; 3 Collegium Budapest, Institute for Advanced Study, Szentháromság u. 2, H-1014 Budapest, Hungary; 4 Parmenides Centre for the Study of Thinking, 14a Kardinal Faulhauber Strasse, D-80333 Munich, Germany

## Abstract

**Background:**

The remarkable potential of recent forms of life for reliably passing on genetic information through many generations now depends on the coordinated action of thousands of specialized biochemical "machines" (enzymes) that were obviously absent in prebiotic times. Thus the question how a complicated system like the living cell could have assembled on Earth seems puzzling. In seeking for a scientific explanation one has to search for step-by-step evolutionary changes from prebiotic chemistry to the emergence of the first proto-cell.

**Results:**

We try to sketch a plausible scenario for the first steps of prebiotic evolution by exploring the ecological feasibility of a mineral surface-bound replicator system that facilitates a primitive metabolism. Metabolism is a hypothetical network of simple chemical reactions producing monomers for the template-copying of RNA-like replicators, which in turn catalyse metabolic reactions. Using stochastic cellular automata (SCA) simulations we show that the surface-bound metabolic replicator system is viable despite internal competition among the genes and that it also maintains a set of mild "parasitic" sequences which occasionally evolve functions such as that of a replicase.

**Conclusion:**

Replicase activity is shown to increase even at the expense of slowing down the replication of the evolving ribozyme itself, due to indirect mutualistic benefits in a diffuse form of group selection among neighbouring replicators. We suggest possible paths for further evolutionary changes in the metabolic replicator system leading to increased metabolic efficiency, improved replicase functionality, and membrane production.

## Background

The majority of recent theories on prebiotic evolution agree that even the most primitive forms of life must have been cellular, and that the first autonomous protocells must have included at least three interacting subsystems: *metabolism, genetic system and membrane *[1-3]. Though we know that these three components are all indispensable for a recent cell to be viable, the assembly of the first complete protocell including all the three subsystems is very unlikely to have abruptly happened from scratch. It is much more probable that the components had been co-opted one by one into a lineage of "pre-protocells" during prebiotic evolution. However, the historical order of the subsystems' appearance and integration is a debated topic among students of prebiotic evolution. According to Luisi [4] and Segré *et al. *[5] the *container/boundary subsystem *(i.e., a primitive membrane) may have been the basis of protocell evolution, and the other two components were built into primitive membrane vesicles later. The vesicles are assumed to be composed of amphipathic molecules with weak to moderate catalytic activities of different specificities. The vesicles could grow at a rate dependent on their actual lipid composition by incorporating new lipid molecules. The better the lipid composition (in terms of how efficiently it drags in lipids from the medium), the faster it grows and divides, i.e., the higher its fitness [4-6]. The snag is with the heredity and thus with the evolvability of these assemblies: neither the lipid building blocks nor the assemblies as a whole do template-copy themselves, which leaves wide open how far they can be units (and subjects) of Darwinian evolution.

The second, maybe more plausible, hypothesis is the "*metabolism first" *scenario in which small molecules (intermediates) play the main role. According to the hypothesis a random set of small organic molecules were the first to appear in the atmosphere and in the prebiotic oceans, and these were then self-organized to form specific reaction networks [7] of a primitive, autocatalytic metabolism. Several classical experiments [8-12] suggest that the spontaneous genesis of organic compounds must have happened on the primordial Earth, but whether the reactions could be spontaneously channelled into a real metabolic system in the absence of specific catalysts is a serious question. The catalytic role of mineral surfaces (e.g. pyrite, montmorillite, calcite) has been invoked [13-17] as a possible solution, but the problem of probably insufficient catalytic specificity on such surfaces is still a serious argument against the "metabolism first" hypothesis. This is where the third prebiotic story – that of the "RNA world" may help.

The "*RNA World*" (or better put: "*Enzymatic Replicator World*") can roughly be classified as a "*genetics first*" scenario, even though the RNA-like enzyme (ribozyme) actors in this play are assumed to have a catalytic function as well, besides their obvious genetic role [18,19]. It is exactly this dual nature of RNA(-like) replicators that render them the best candidates for the role of booting up life: they are evolvable genetic units, but they also feature metabolic phenotypes that are dependent on their actual genetic information content.

Elementary combinatorics suggests that there can be three so-called infrabiological' systems [20] consisting of any two of the three aforementioned systems. Qualitatively different, coupled autocatalytic systems are the exciting terrain of the emerging field of 'systems chemistry' [21]. The present study investigates an example of the metabolism-template (MT) doublet. Interestingly, a similar doublet was originally considered by Gánti [22] to be the minimal chemical supersystem for life: the boundary was added later to the model [23].

The most serious difficulty with the ribozyme world scenario is ecological in nature: different replicators occupying the same habitat and replicating with different rates will compete for the (common) resources of their replication. Ultimately the fastest one will exclude all the others, which means a dramatic decrease of genetic diversity within the population of replicators, for two reasons: first, because all but the winner's information is eliminated from the system; second, because the winner is the one that replicates fastest, and the fastest replicator is most probably a very short polymer, with a limited information content [24]. In the prebiotic evolutionary context, all these mean the loss of most of the genetic information carried by any diverse replicator population. However, a working protocell had to maintain a reasonably high amount of genetic information to accomplish many phenotypic tasks (i.e., encode many different enzymes). That amount of information also had to be reliably passed on to their offspring to maintain their functionality through many generations.

The problem of maintaining genetic information under prebiotic conditions can only be solved on the basis of some higher-level control of the – otherwise inevitable – competitive exclusion process. The first steps in this direction were taken by Eigen [24]; see also Eigen and Schuster, [25]. Their hypercycle model assumes that a set of self-replicating ribozymes provide each other with heterocatalytic help in a circular topology (i.e.: replicator A supports B supports C supports A). The hypercyclic coupling of the replicators automatically maintains coexistence even if the replication rates of the hypercycle members are different – as long as no mutations pop up in the system. However, the hypercycle model has been proven to be vulnerable against parasitic mutant replicators: both "selfish" (accepting but not providing replication support) and "short circuit" (supporting a farther member of the system) parasites ruin hypercyclic coexistence and lead to the ultimate exclusion of all but a single replicator [26]. The only way the hypercycle can be saved is by compartmentalization: the system has to be wrapped into membrane vesicles, and selection on the level of the vesicles can maintain the coexistence of hypercyclically coupled replicators [27]. Although it has been proposed that hypercycles may 'jump to life' when their constituents are absorbed to mineral surfaces [28] by virtue of the infolding mesoscopic (rotating spiral) patterns, which make them resistant to parasites; this model has been shown to be unstable under the assumption of a 'patchy environment' in two dimensions [29].

Our approach is different from the hypercycle model in that we assume a set of ribozyme replicators catalysing a network of chemical reactions (metabolism: see ref. [30]), which in turn produces monomers for the replication of the ribozymes themselves. That is, the ribozymes contribute the production of the common resource (through the metabolic network) by their specific catalytic activity, and metabolism aspecifically contributes to ribozyme replication by providing monomers. We confirm that this system is viable (i.e., coexistent) without a membrane envelope, it keeps the abundance of parasitic mutants low, and that the coexistent parasites of the system can serve as pre-adaptations for subsequent evolution into ribozymes of potentially great utility for the metabolic system as a whole. Specifically, in this study we explore the novel case of parasite evolution towards a replicase function, but we also sketch a few different routes of possible further adaptations. Thus our present work bridges the gap between the metabolic model of Czárán & Szathmáry [30] that had no replicase in it and the replicase model of Szabó et al. [31] that had no metabolism.

## Method

### The model

#### 1. The metabolic model without replicase

**Figure 1:**
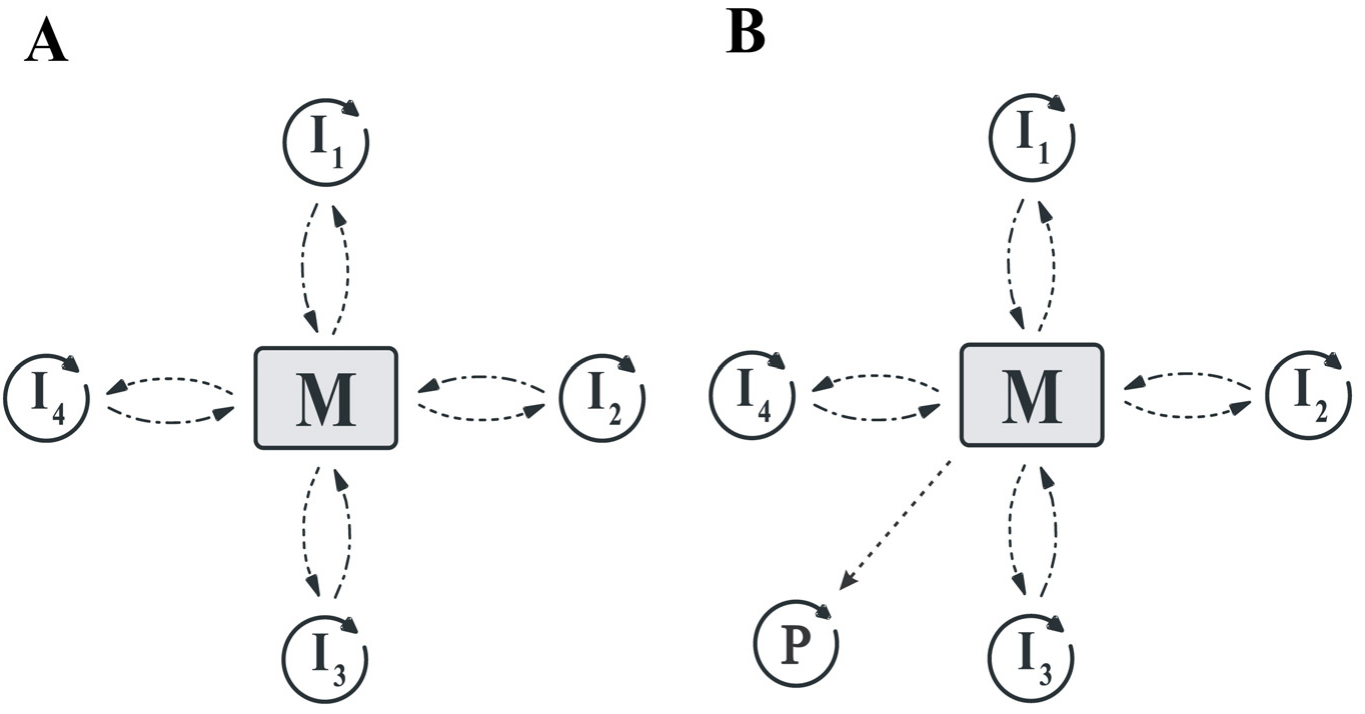
**The metabolic system. ***a: I*_1..4 _are the metabolic replicators; *M *is metabolism. Dashed arrows represent the supply of metabolites (monomers) used for replication; solid circular arrows show replication; dashed-dotted arrows mean the catalytic effect of metabolic replicators helping metabolism. *b: *Parasitisation of the metabolic system: the parasitic replicator *P *uses the monomers supplied by metabolism but it does not help producing them.

Czárán and Szathmáry [30] consider a number of relatively short replicator macromolecules, each of which is capable of catalysing one (and only one) essential reaction of a metabolic reaction network M. Metabolism is specified neither in stoichiometric nor in topological terms; it is assumed that Mproduces the monomers for the replication of the macromolecules themselves, and that the catalytic help of the replicators is essential for monomer production. In other words: metabolism aspecifically supports the replicators by providing them with monomers, and the replicators specifically support metabolism by catalysing certain reactions of it (Figure 1a). The extinction of any one of the replicators I_i_results in the collapse of metabolism and thus the demise of the whole replicator system. The simplest mathematical model for the temporal dynamics of a nonspatial system with these properties is

(1) $$\begin{array}{cc} \frac{dx_{i}}{dt}=x_{i}\left[k_{i}\cdot M\left(x\right)-\phi \left(x\right)\right], & \left(i=1,...,n\right) \end{array}$$

where *x*_*i *_is the concentration, *k*_*i *_is the growth rate of replicator *i*. *M*(x) expresses the effect of metabolism on the replication rate. It is a common multiplicative function of the replicator concentrations x, so that each replicator type needs the presence of all the others to be able to replicate, but the metabolic help received is aspecific. *φ *is an outflow function acting as a concentration dependent selection constraint, keeping the total concentration of the replicators constant. Although all replicators must be present for *M *to be positive, we know that this does not preclude competitive exclusion of all other types by the fittest (of largest *k*_*i*_) replicator [25]. Since *M *is the same in all equations of [Eq.1], even very small positive concentrations of the competitively inferior types maintain the advantage of the dominant in terms of the speed of replication. Therefore the replicator of largest *k*_*i *_will always multiply the fastest of all, excluding the slower (of smaller *k*_*i*_) ones, and with the excluded type missing from the metabolic network the system ultimately collapses with all replicator types going extinct. That is, the metabolic model is not capable of maintaining the community of replicators – at least not in a well-mixed medium.

Czárán and Szathmáry [30] show that the very same metabolic system is robustly persistent and coexistent in a spatially explicit cellular automaton model even if the replication rates of the replicators are different (Appendix gives details of the Czárán-Szathmáry model). The crucial assumption of the spatial model is that any replicator type needs the presence of all the other types within a small section of space (called the *metabolic neighbourhood*) around itself for being able to replicate, because local monomer supply depends on local synthesis – diffusion cannot deliver monomers at sufficient concentrations to longer distances. Thus the somewhat surprising result of stable coexistence in the cellular automaton is due to the effect of higher-level selection for metabolically complete replication neighbourhoods within small sections of space. Rare replicators are at a relative advantage compared to common ones, because they have more chance to be complemented by all the common types within a small metabolic neighbourhood – more common replicators have less chance to find at least one rare type molecule nearby and thus to get copied.

Besides its persistence and coexistence in itself, the spatial metabolic model has been shown to resist parasites. A parasitic replicator of the metabolic system is one that uses the monomers provided by metabolism for its own replication, but does not itself contribute to monomer production at all (Figure 1b). Such parasites are unable to kill off the spatial metabolic system even if their replication rate *k*_*p *_is much larger than those of the cooperating replicators. The simple reason for this is that wherever the parasite becomes abundant, the metabolic system breaks down locally, therefore any further parasite replication becomes impossible, while neighbourhoods devoid of parasites still produce the cooperating replicator types. The overall effect of this spatial regulation is that the parasite coexists with the metabolic system, albeit at a relatively low frequency. In a considerably large part of the parameter space of the model the parasites are definitely not able to ruin the metabolic system [30].

#### 2. The metabolic model with an evolving replicase

**Figure 2:**
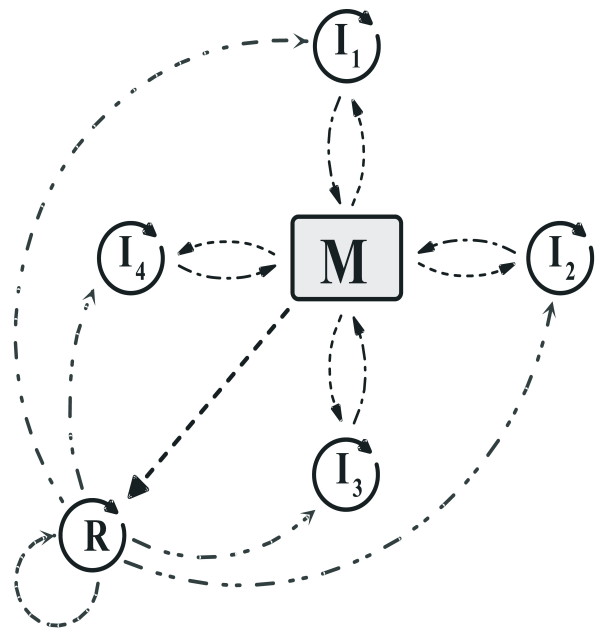
**Metabolic system with a parasite evolved to the replicase function. **The dashed-double-dotted lines represent the sequence-aspecific replicase activity of the converted parasite.

Due to the specific enzymatic roles they play, metabolically cooperating replicators are rather strictly constrained in changing their spatial structure and thus also their monomer sequence. This does not apply to parasites lacking any catalytic function, however. Once the parasites are around, neither doing real harm to the metabolic system as a whole, nor going extinct, they are free to mutate. Mutants may be even more harmful than the original parasite they are derived from, they may be neutral compared to their "parents", or they may obtain traits that are of help for the survival of the metabolic system – we shall discuss the latter possibility in more detail in Discussion. For now it is sufficient to note that harmful mutants are quickly eliminated from the system by the very same mechanism that keeps the original parasite rare: they kill off nearby metabolic replicators, themselves committing suicide this way. Therefore, harmful mutants have no chance to disperse – they behave like hyper-virulent virus strains killing their host before it could reproduce and spread them. What we shall explore in more detail is the case when the parasite evolves to a beneficial function for the metabolic system by gaining replicase activity (Figure 2).

Template replication and catalytic activity are antagonistic in the sense that replication is fastest on unfolded, short and linear templates whereas catalytic activity requires that the polymer be long and folded into relatively compact and stable spatial (secondary and tertiary) structures. This means that a good template is unlikely to be a good catalyst at the same time. To take this plausible inverse relation of replication rate and catalytic activity into account in a metabolic system infested with mutable parasites we have extended the Czárán-Szathmáry model [30] (see also the Appendix), as follows:

##### Mutations

Parasites replicate and decay exactly the same way as metabolically cooperating replicators do. The difference between cooperators and parasites is that a) the parasites are not needed for the replication of any other template (because they do not contribute to monomer production), b) they can mutate whenever they replicate, and c) the mutants may occasionally obtain some replicase activity. Specifically, mutation affects two crucial traits of parasites: their replication rate *k*_*p *_and their aspecific replicase activity *r*_*p*_. These traits are in a trade-off relation one with the other: if a mutation happens to increase the replication rate of a parasite, it will decrease its replicase activity, and *vice versa*.

**Figure 3:**
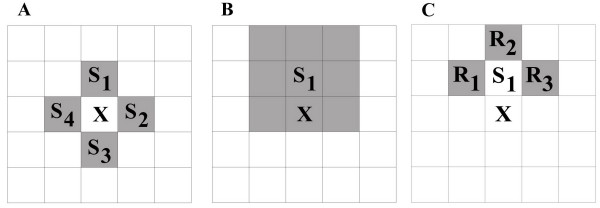
**Neighbourhoods in the model. ***a: *The replication neighbourhood. *X *is an empty site; its four orthogonal nearest neighbour sites (von Neumann-neighbourhood) constitute the replication neighbourhood (grey), occupied by replicators *S*_1..4_. *b: *The metabolic neighbourhood. *X *is an empty site; *S*_1 _is a replicator on one of the four orthogonal nearest neighbour sites of the empty site. The metabolic neighbourhood of *S*_1 _is the rectangular subgrid (in this case the Moore-neighbourhood) centred on *S*_1 _(in grey). Metabolic neighbourhood size is one (*h *= *1*). *c: *The catalytic neighbourhood. *R*_1..3 _are replicases in the catalytic neighbourhood of *S*_1 _(in grey). One of these replicators is drawn to help the replication of *S*_1 _onto the empty site (*X*).

The algorithm of the mutation process is the following: If a parasite is chosen for replication from a replication neighbourhood (Appendix, Figure 3a), we draw a random number (*d*_*k*_) from a Gaussian distribution of mean 0 and standard deviation *σ*_*k*_. *d*_*k *_determines the mutated replication parameter *k'*_*p *_of the „daughter”-parasite according to the

(2) $$k_{p}^{'}=k_{p}\left(1+d_{k}\frac{k_{\max}-k_{p}}{k_{\max}}\right)$$

equation, in which *k*_*p *_is the replication parameter of the „mother” and *k*_***max ***_is the arbitrary upper limit for the replication parameter. To avoid mutations producing nonsense (i.e., negative, or above-limit) replication parameters, [Eq.2] scales the actual value of *k'*_*p *_into the [0, *k*_***max ***_] range for any reasonable (i.e., not unrealistically large) value of *d*_*k *_. Our choice of this specific mutation algorithm is justified not only by practical considerations (i.e., by the need for scaling the phenotypic effects of mutations into a reasonable range). The phenotypic effect of a mutation is likely to depend on the actual trait value: it is realistic to assume that the quantitative effect of the same mutation may be smaller close to the limit of the trait range. This assumption is built into Eq.2 as well.

Once the mutation change *d*_*k *_for the replication parameter is specified, the trade-off relation

(3) *d*_*e *_= -*a*·*d*_*k*_

determines the expected value of the mutation change in replicase activity, *d*_*e*_. *a *is the trade-off parameter – the larger it is, the more severe the trade-off between replicase activity and reproduction rate. To allow for some „wobbling” in the trade-off relation, we add a Gaussian noise term ξ(0, *σ*_*r*_) of 0 mean and *σ*_*r *_standard deviation to the *d*_*e *_value, to obtain the actual replicase activity parameter *d*_*r*_:

(4) *d*_*r *_= *d*_*e *_+ ξ(0, σ_*r*_)

**Figure 4:**
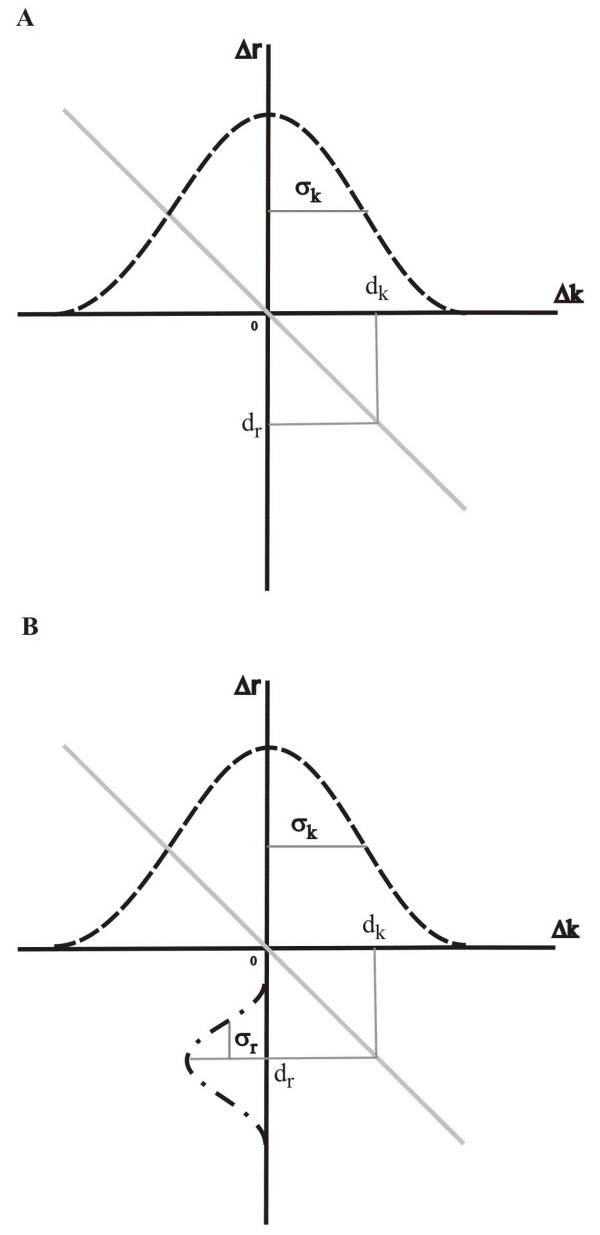
**The replicase activity – replication rate trade-off. ***d*_*k *_is the change in *replication rate *due to a single mutation. *d*_*k *_is randomly drawn from a Gaussian distribution (dashed line) of mean 0 and standard deviation *σ*_*k*_. *d*_*r *_is the change in *replicase activity *due to the same mutation. The solid line is the trade-off curve of parameter *a. *In panel A *d*_*r *_is simply calculated from the *d*_*r *_= - *a d*_*k *_linear trade-off function (hard trade-off case). In panel B a noise component of Gaussian distribution (dashed-dotted line) with mean 0 and standard deviation *σ*_*r *_is added to *d*_*r *_to allow for some "wobbling" in the trade-off relation (soft trade-off). *σ*_*r *_is the *plasticity *parameter of the trade-off.

*σ*_*r *_specifies the plasticity of the trade-off relation – the larger *σ*_*r *_the softer the trade-off, i.e., the more the relation of the two parameters can deviate from the trade-off line [Eq.3]. *r'*_*p*_, the actual replicase activity of the mutant „offspring” is determined using *d*_*r*_, *r*_*p *_and *r*_***max ***_by rescaling in a way completely analogous to [Eq.2]. Figure 4 illustrates how *d*_*k *_and *d*_*r *_are calculated using the trade-off function. We have applied the reflective boundary condition on the margins of the trait range.

The „daughter-copy” of the parasite is the mutant, whereas the „mother-copy” keeps its original phenotype (i.e., replication rate and replicase activity).

##### Replicase support

A parasitic replicator *R *either provides help for the replication of neighbouring template molecules or it hampers template replication, depending on its actual replicase activity *r*_*r*_. If *r*_*r *_exceeds 1.0 (the basic catalytic activity of the hosting surface), then *R *acts as a real replicase, i.e., it helps replication, but a parasite with *r*_*r *_< 1.0 acts as a „poison” for surface catalysis: it binds the template and does not even let the mineral surface help the replication process. Replicase *R *that supports (or suppresses) template *S *is selected at random from the catalytic neighbourhood of *S *(Figure 3c).

If there is no replicase around then reproduction takes place at the basic rate (of value 1.0) provided by the (e.g., pyrite) surface itself. Of course a parasite (replicase) molecule cannot help its own replication; it needs another replicase in its catalytic neighbourhood for that.

##### Updating and diffusion

The cellular automaton is updated one randomly chosen site at a time. If the site chosen is occupied by a replicator, it becomes empty with a probability *p*_*d*_. If it is empty, then all the replicators (metabolic and parasitic) in its replication neighbourhood (Figure 3a) compete for occupying the empty site with a copy of themselves, according to the stochastic rules [Eqs.5–8] in Appendix. One generation consists of a number of such updates equal to the number of sites (90.000) in the lattice, so that each site is updated on average once per generation.

The diffusive movement of replicators is modelled by the algorithm of Toffoli and Margolus [32], which preserves particle number and frequency distribution within the grid. The intensity of diffusive mixing depends on the number of diffusive steps per generation (*D*) taken by a replicator on the surface.

## Results

### 1. Coexistence and parasite resistance

With *σ*_*k *_= 0.0, i.e., with parasite mutation banned, the model is identical to that of Czárán and Szathmáry [30]. The main conclusions of the non-mutable cellular automaton model are that

a) the surface-bound metabolic system remains persistent in a large part of its parameter space, even if the replicators have different replication rates;

b) diffusion does not ruin persistence – to the contrary, mixing is a necessary component of coexistence, because large monotypical patches of the same replicator type are doomed to extinction;

c) parasites are kept in check by the cooperating replicators, through their inevitable need for monomers which can be supplied to the parasites only by the cooperators, therefore even parasites of high replication rates are suppressed to a low frequency in the system.

A more detailed treatment of the results obtained from the non-mutable model is given in Appendix.

### 2. Replicase evolution: direct and indirect selection

**Figure 5:**
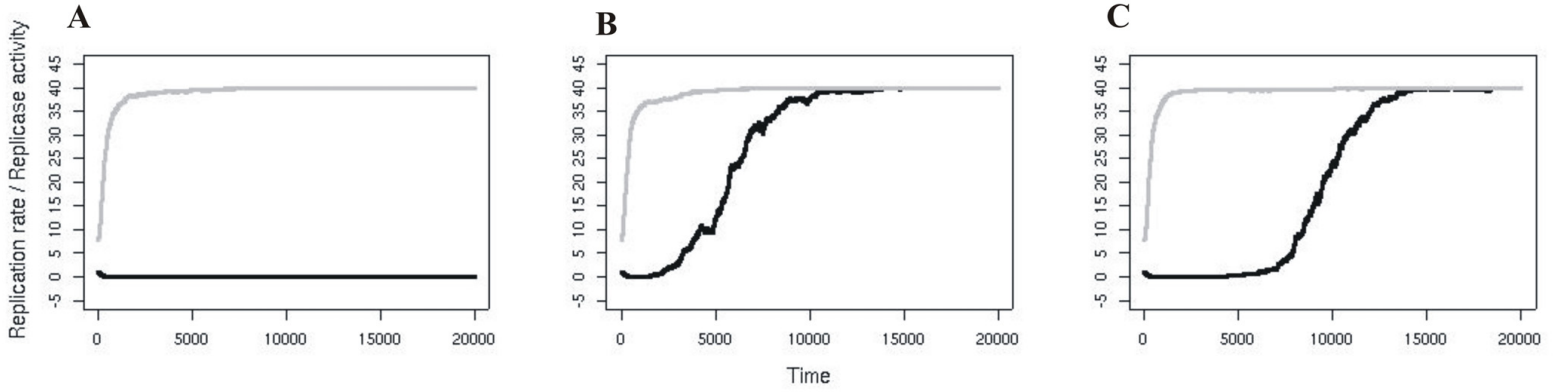
**Evolution of replicase activity. **Change of replicase activity (black) and replication rate (grey) for hard and soft trade-off. Fixed parameters: grid size: *G *= 300 × 300; system size: *n *= *4*, of which one is a mutable parasite; *k*_*max *_= *r*_*max *_= *40*; length of simulations: *t*_*max *_= 20.000; empty site claim: *C*_*e *_= *2.0*; decay rate for all replicators: *p*_*d *_= *0.2*; replication rates *k*_1 _= *2.0*, *k*_2 _= *4.0*, *k*_3 _= *6.0 *and *k*_4 _= *8.0 *(parasite). a: hard trade-off: *h *= 1, *D *= 4, *σ*_*r *_= 0.0, *a *= 1.0. b: soft trade-off: *h *= 1, *D *= 4, *σ*_*r *_= 0.1, *a *= 1.0. c: The same as b with faster replicator diffusion:*h *= 1, *D *= 20, *σ*_*r *_= 0.1, *a *= 1.0.

By allowing for mutation changes in the parasite according to trade-off relations [Eqs. 2–4], evolution either towards higher replication rates alone, or to both higher replication rates and a subsequent increase in replicase activity, occurs (Figure 5). Which of the two comes about depends on the strength and the plasticity of the trade-off between replication speed and replicase activity. Too strong and too hard a trade-off – i.e., large *a *in [Eq.3] and small *σ*_*r *_in [Eq.4] – means that a small increase in replication rate causes a large decrease in replicase activity. Since higher replication rates have a direct positive effect on fitness, the system evolves towards increasingly efficient parasites, but steep slope and the small wobbling of the trade-off relation do not give a chance for replicase activity to catch up even when replication rate has reached its maximum (Figure 5a).

In contrast, smaller *a *and/or higher *σ*_*r *_parameters (i.e., weaker and/or softer trade-off relations) result in a fast increase in replication rate first, followed by a slower increase in replicase activity later (Figure 5b, c). The pattern and the timing of these evolutionary changes can be explained in terms of direct and indirect selection forces acting on the parasites of the metabolic system.

The more obvious case is direct selection acting in favour of faster reproduction: a larger replication rate is clearly of a direct fitness increasing effect – this is the cause of the steep increase in average replication rate among parasites. However, replicase activity itself has no direct effect on the fitness of the parasite, because parasite molecules with a higher replicase activity cannot help their own replication, only that of their neighbours. In spite of this we find slower, yet steady evolution towards increased replicase activity *r*. The reason for this is indirect selection: better replicase molecules increase the fitness of their neighbours. The beneficiary neighbours are either metabolic replicators or parasites. With limited spatial dispersion of the offspring, neighbouring parasites are with a good chance „relatives” of the one that gives catalytic help: what we see is a typical case of kin selection at work. This in itself results in a fitness increase for parasites with a higher replicase activity in a poorly mixed system, but there is also another indirect beneficial fitness effect which does not require limited spatial mixing. Metabolic neighbours reciprocate the catalytic help they get from the replicase, by providing monomers for reproduction of the latter. An efficient replicase has more chance to be surrounded by a complete set of metabolic replicators (and thus being copied) simply because the local density of replicators around it is higher than around less efficient ones. This indirect positive selection effect does not depend on spatial mixing (i.e., on diffusion), and even if so, it definitely does not require slow (or no) diffusion.

**Figure 6:**
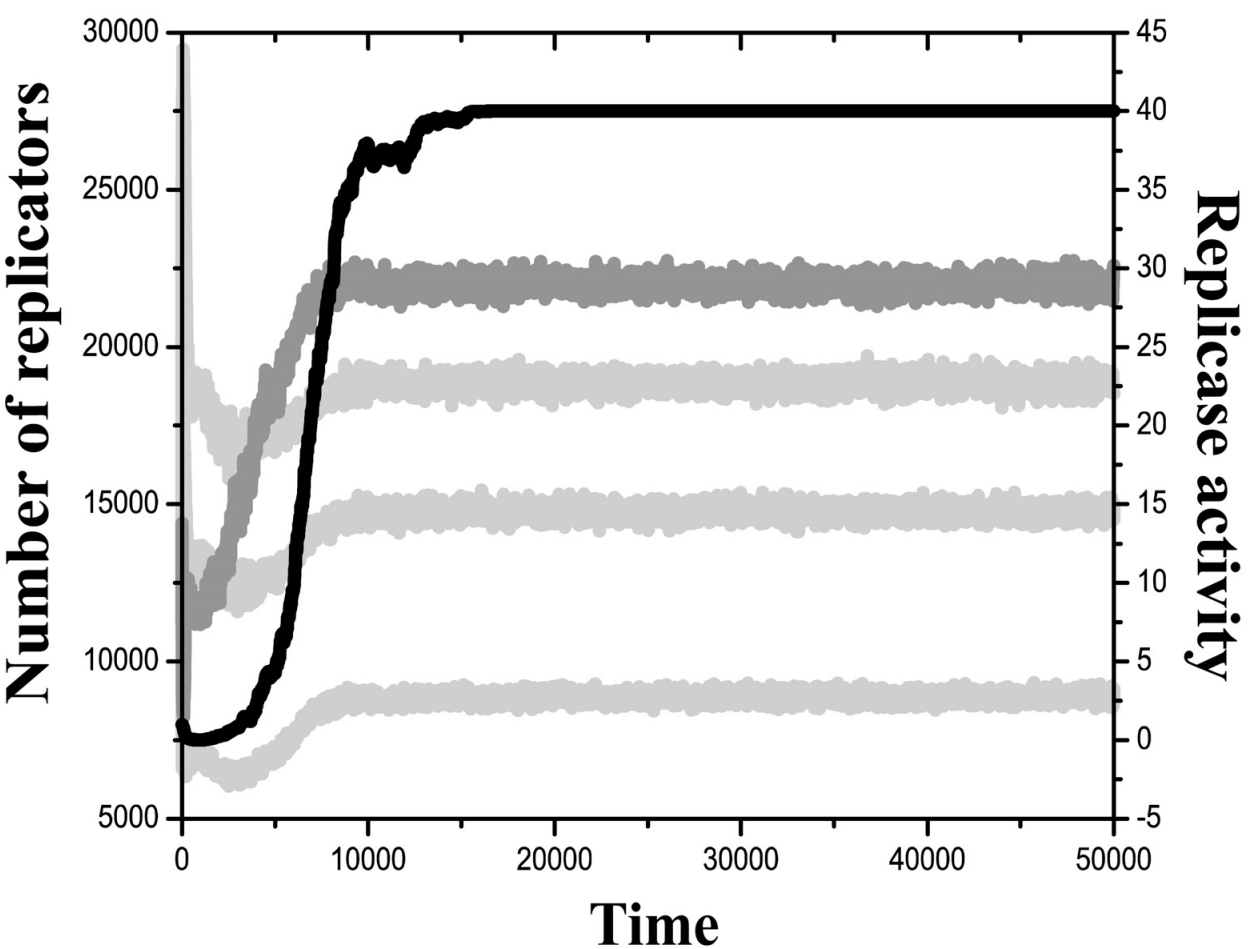
**The effect of an evolving replicase replicator on the metabolic system. **Light grey lines: metabolic replicators; dark grey line: the parasite (replicase) replicator; black line: replicase activity. *h *= 1, *D *= 4, *σ*_*r *_= *0.05*, *a *= 1.0. All other parameters as in Figure 5.

As for the real influence of diffusion rate *D *on replicase evolution, we have performed simulations with different diffusion intensities (up to *D *= 20) and found no qualitative difference compared to lower rates of diffusion (Figure 5b, c). A slight improvement in the overall performance of the system is observed in the course of replicase evolution: the total number of replicators increases at the expense of empty sites. As the parasite converts into a "replicase" and starts working for the common good, it becomes released from the check by metabolic cooperators and can reach high abundance in the system (Figure 6).

## Discussion

### 1. Weak altruism and group selection in the metabolic replicator model

It is important to consider our model from the point of view of models of group selection. First, we are dealing with an MLS1 (multi-level selection of type one: [33,34]) in which the only focal units are the replicators. In contrast, the stochastic corrector model is of the MLS2 type since both the replicators and the groups are focal units in the process. Second, the previous metabolic model [30] and the replicase evolution model behaved entirely differently when the rate of diffusion was increased: the former survived and the latter collapsed. Why is this so? The answer is, we propose, that replicators better aiding metabolism are weak altruists, whereas better replicase replicators are strong altruists, as can be checked by the mutation test of Nunney [35]. For both types of altruist it holds that *within the same group *the altruist has smaller fitness than the selfish variant. The mutation test compares *different groups *in that it asks: would a selfish unit when mutated into an altruist in a group have an absolute loss in fitness? For weak altruists such a drop is not realized because by contributing to the benefit of the group there is a net gain in absolute fitness for the altruist, even though within the same group it has smaller fitness relative to the selfish types. Replicators aiding metabolism can 'see themselves', or 'scratch their own back' as it were, hence they can qualify as weak altruists. In contrast, as mentioned before, replicase replicators cannot see themselves: another copy is needed for helping the focal individual, thus they are strong altruists. It is known that weak altruism can spread by random group assortment whereas the spread of strong altruists need positive assortment (e.g., [34]). This is the reason why the metabolic model failed to collapse under increasing diffusion: it just converged to a trait group model with random assortment of the kind analyzed by Szathmáry [36]. In contrast, limited diffusion was mandatory for survival of the replicase model, which created positive association.

If all this holds, why do we find then that in our combined model the replicase evolved despite increasing diffusion rate? The answer has been given above by the term 'indirect selection': through the effect on metabolic replicators, even one replicase can 'see itself' (cf. Figure 5 – faster diffusion does not harm replicase evolution). This shows that systems chemistry will be an exciting field for evolutionary theory as well.

### 2. Metabolic parasites as preadaptations

As briefly mentioned in part 2 of the Results section, metabolic parasites can mutate to different functions. Some of the mutants might be even more harmful than just a non-cooperating parasite, directly damaging the metabolic system – but these mutants are doomed to fast extinction, because they kill their „hosts” (the cooperating replicators) before they could enjoy the benefits of having them around, and thus they die out themselves too. Most mutants will be neutral, i.e., just as parasitic as their ancestors, doing no more and no less harm to the system than just tapping the metabolism for monomers and using them for their own replication. Neutral mutants will diversify and thus "scan" the sequence space, and they will all coexist with the hosting system just as their ancestors do. This means that many neutral mutants of different sequences accumulate within the metabolic system as new mutations occur. Finally, some of the many neutral mutants might mutate to something that potentially carries some utility for the metabolic system itself – and that something might be many different things.

We have explored the case when mutants can show a little better replicase activity than the very basic catalytic help to replication given by the mineral surface harbouring the metabolic system (Figure 2). This has been shown to indirectly benefit the mutant itself, and obviously it is positively selected at the level of the whole metabolic system too, because it increases the replication rates of all the replicators present. Thus, the replicase will spread, and the relative fitness of the mutant system, compared to one with a weaker replicase activity of the „converted” parasite, increases.

**Figure 7:**
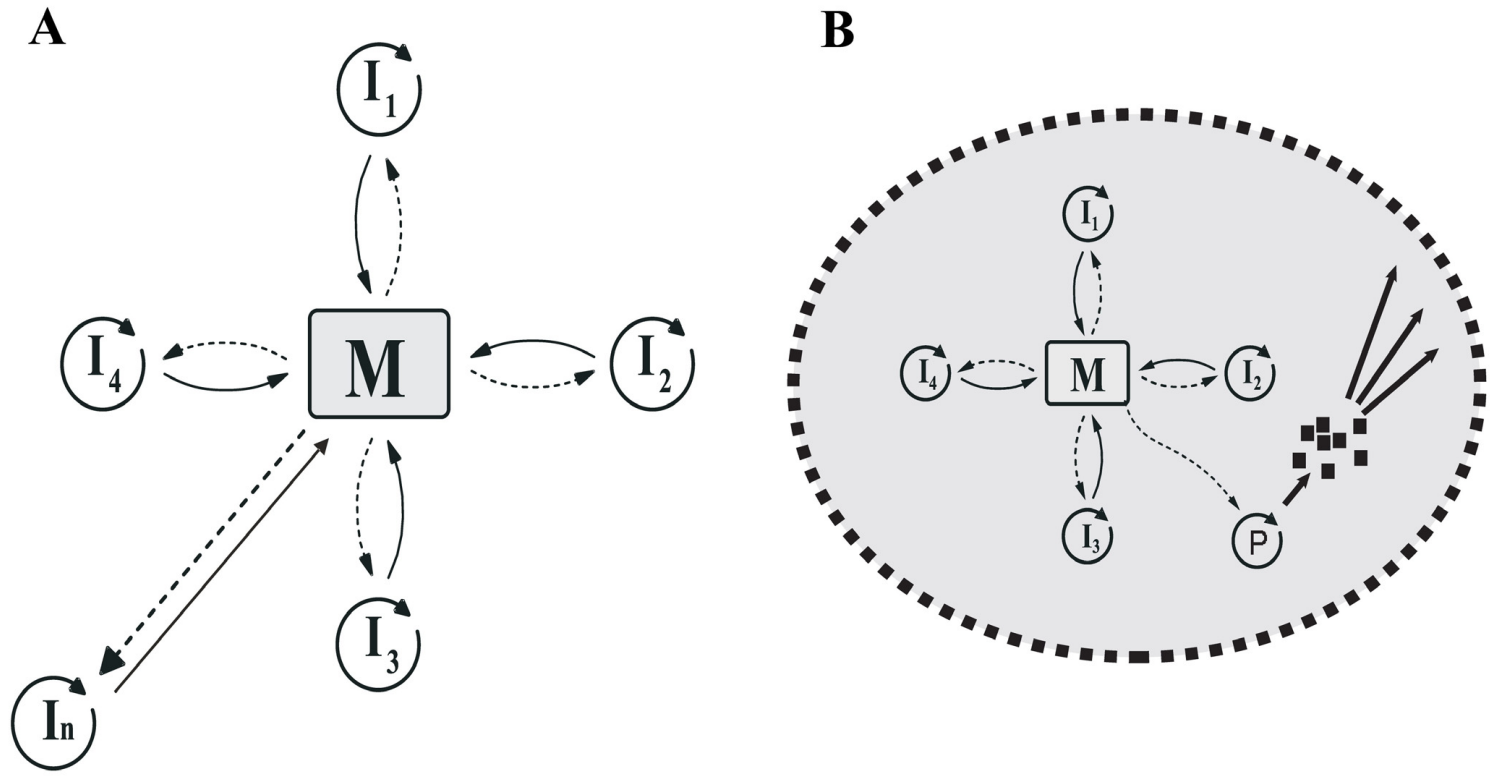
**Hypothetical metabolic systems**. A parasite could evolve for a new metabolic function (a) or to catalyse a terminal reaction of metabolism that produces membrane units (b).

Some other mutants may be useful in the metabolic reaction network, possibly catalyzing one or another reaction better than the previous replicator, or even opening new and useful reaction routes. In any case the new, more efficient mutant spreads, and the metabolic system itself also benefits (Figure 7a).

Still other mutants may open yet another metabolic route by converting some of the intermediate metabolites or waste compounds to small amphipathic molecules. These may become the first building blocks of a primitive membrane structure wrapping the metabolic system into small vesicles. Such a mutant would be of high selective value to the whole replicator community, because it would result in the encapsulation of the – thus far surface-bound – metabolic system, providing it with a more efficient method for group selection: the stochastic corrector mechanism [37] (Figure 7b).

All in all, the metabolic parasites of the surface-bound metabolic system may represent preadaptations to virtually any possible catalytic function in a future protocell, including better metabolic enzyme functions, membrane production, and replicase activity. Membrane production would ultimately lead to the „prebiotic takeoff” of the metabolic system, by detaching it from the surface allowing it to enter a new environment and a more efficient selection regime in a proto-cellular structure (cf. the 'abstriction from the surface' scenario of [38]). All these benefits depend critically on the continuous input of mutations of metabolic parasites on a hosting surface – and these circumstances seem inevitably to be present in the spatial metabolic system anyway. We have explored in some detail the case when mutants gain replicase activity, but both the adoption of some mutants as new metabolic replicators by the metabolic system and the hypothetical case of 'abstriction' require detailed modelling for more theoretical support.

Our approach is similar to the proposed scenario of Ma *et al. *[39] who were interested in the *de novo *appearance of a single type of nucleotide synthetase on a surface, following the spirit of the model of Szabó *et al. *[31] for the appearance of replicases. They envisaged the subsequent appearance of a ribozyme synthesizing membrane components, and the appearance of a replicase only after membrane compartmentation. We have shown here that the emergence of a replicase within a ribozyme-catalyzed surface-bound pre-existing metabolic system is more likely than the appearance of a synthetase just by itself as in [31]: this is due to the favourable indirect selection for better replicases by feedback through the metabolic system. Emergence of the replicase by itself requires very limited diffusion; the system presented here is free of that constraint, but of course the metabolites must stay in a limited neighbourhood of their production, as also assumed in [39].

## Conclusion

In this paper we concentrated on dynamical coexistence of useful replicators with passive population structure. While resistance against parasites can be provided by couplings different from those in our model (e.g. in [40,41]), we are convinced that focusing on metabolism is especially important because it points in the direction of interesting complexification leading towards the origin of cells with templates, membrane and metabolism.

## Appendix

### The non-mutable metabolic replicator system

The basic metabolic machinery is the same cellular automaton as the one described in Czárán and Szathmáry [30]: it consists of a 300 × 300 square grid of sites with a toroidal topology to avoid edge effects. Each site of the grid can contain at most one replicator molecule, which may be either one of the metabolic „enzymes” (cooperating replicators) or a parasite (a replicator without a metabolic function).

### Replication and decay

Besides catalysing specific reactions of metabolism, metabolic replicators can do two things: replicate, or decay. For a replication event of any template *s *into a neighbouring empty site to occur, *s *must be complemented by all the metabolically active types present in its metabolic neighbourhood of a certain size (Figures 3a, b). Note that the parasite requires all the *n *metabolic types around for its replication, but the metabolic replicators do not need the presence of the parasite in their metabolic neighbourhood for their reproduction.

The claim of template *s *to replicate into an adjacent empty site is:

(5) *C*(*S*) = *r*_*s *_· *k*_*s *_· *M*_*s*_,

where *r*_*s *_is the replicase support (of fixed value 1.0 in the non-mutable model; may be different in the mutable system – see main text), *k*_*s *_is the reproduction rate and *M*_*s *_is the metabolic support for replicator *s*.

(6) $$M_{s}={\left[\prod_{i=1}^{n}f\left(i\right)\right]}^{1/n},$$

in which *f(i) *is the copy number of metabolic replicator type *i *within the metabolic neighbourhood of *s*. *n *is system size (the number of metabolic replicator types). Observe that the parasite, which is type *n *+ 1, is not counted here. Notice also that *M*_*s *_is proportional to the geometric mean of within-neighbourhood metabolic replicator frequencies – if any one of the *n *metabolic replicator types is missing from the metabolic neighbourhood, then *M*_*s*_, and thus also *C(s)*, is zero. The chance of *s *to replicate into the empty site is:

(7) $$P_{s}=\frac{C\left(s\right)}{C_{e}+\sum_{l}C\left(l\right)}$$

where *l *are the four orthogonal nearest neighbours (the replication neighbourhood, Fig. 3a) of the empty site, and *C*_*e *_is the claim of the empty site to remain empty. Thus the probability that the empty site remains empty is:

(8) $$P_{e}=\frac{C_{e}}{C_{e}+\sum_{l}C\left(l\right)}$$

Decay is aspecific, defined as a constant probability *p*_*d *_for any occupied site to become empty in time *t *+ 1, irrespective of what type of replicator it harboured in time *t.*

### Results of the non-mutable metabolic replicator model

The most important result with this setting is that the cellular automaton is capable of producing coexistence in a large part of its parameter space. No conspicuous mesoscopic patterns like monotypical patches or spiral waves [28] arise in any simulation, since a relatively homogeneous spatial distribution of the metabolic replicator types is necessary for many neighbourhoods to contain a metabolically sufficient set of macromolecules and thus for the replicators to survive. That is, a persistent system cannot show an aggregated pattern.

**Figure 8:**
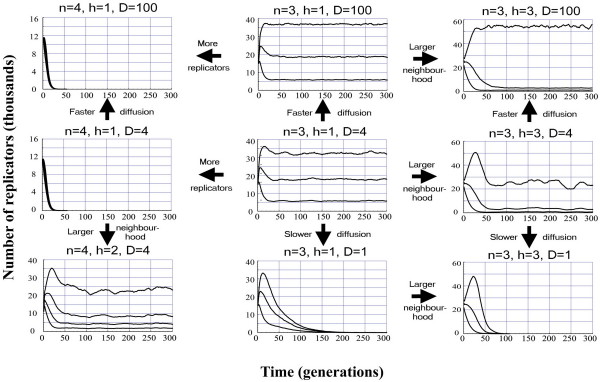
**Time series of replicator abundances. **Temporal change in the numbers of cooperating replicators in the metabolic system without the parasite, for different parameter sets. Fixed parameters: grid size: *G *= 300 × 300; length of simulations: *t*_*max *_= 300; empty site claim: *C*_*e *_= *2.0*; decay rate for all replicators: *p*_*d *_= *0.2*; replication rates *k*_1 _= *2.0*, *k*_2 _= *4.0*, *k*_3 _= *6.0 *and (wherever applicable)*k*_4 _= *8.0*. Lines represent the abundances of the different types of metabolic replicators, the fastest (of largest *k*_*i*_) being the most abundant.

From the viewpoint of template coexistence, the most relevant parameters of the model are diffusion rate (*D*) neighbourhood size (*h*) and system size (*n*). Figure 8 illustrates the effects of these parameters on the ecology of the metabolic system without parasites and mutation.

#### Diffusion

Increased diffusion rate promotes coexistence in any case of sufficient replicator density. However, very sparse systems are killed by fast diffusion, because potentially cooperating replicators are dispersed apart in space and thus they have little chance to be metabolically complemented. Diffusion helps parasites in spreading, but they cannot drive the metabolic system extinct even at very high diffusion rates: the spatial regulation mechanism works well also with fast diffusion.

#### Metabolic neighbourhood size

For a fixed system size, there is an optimum neighbourhood size, below which the chance that it contains a metabolically complete set of replicators is too small or even zero (metabolism "does not fit in") and above which replicators start to "feel" the overall population density, and the results are similar to those of the nonspatial model (see [Eq.1] in the main text). Note that metabolic neighbourhood size corresponds to the distance within which metabolite concentrations do not shrink too low, i.e., indirectly it depends on metabolite diffusion rates.

#### System size

For any fixed combination of neighbourhood size and diffusion rate, increasing system size ultimately leads to the collapse of the system for the reason discussed above in relation to neighbourhood size reduction: neighbourhood size is the absolute upper limit of system size. However, this is partly due to an artificial effect of coarse spatial resolution: assuming more sites within a metabolic neighbourhood of the same physical size, the system size effect could be weaker.

Decreasing system size makes coexistence more likely in any parameter setting, but it is to be noted that we do not consider the absolute efficiency of metabolism to be a function of system size in this model. This would be reasonable to assume, however, since more replicators might catalyse a more efficient metabolism, giving more chance of survival for the larger system. That is, we apply a worst-case assumption here.

### Parasites

Parasite molecules benefit from the presence of metabolic cooperators which drive metabolism (the source of monomers for replication), but they do not themselves contribute to monomer production at all. As in Czárán and Szathmáry [30], we found that a coexistent metabolic system cannot be killed off by such a parasite, even if its replication rate exceeds that of the fastest cooperating macromolecule type. Extremely efficient parasites can reduce the concentration of the metabolically active replicators by simply occupying most of the surface available, but even then local neighbourhoods containing fewer or no copies of the parasite will be at an advantage and thus increase the relative frequency of cooperators. The result is a persistent metabolic system which is coexistent with its parasite. At whichever parameter set the system is persistent without the parasite, it is also persistent with it in most cases. A system that collapses would do so without the parasite as well.

## Authors' contributions

The model was a common idea of KB, TC and ESZ, KB and CT have designed and implemented the metabolic system in the cellular automaton framework. All authors contributed to writing the manuscript and drawing the figures and approved the final version.
